# Scale-up of Kenya’s national HIV viral load program: Findings and lessons learned

**DOI:** 10.1371/journal.pone.0190659

**Published:** 2018-01-11

**Authors:** Matilu Mwau, Catherine Akinyi Syeunda, Maureen Adhiambo, Priska Bwana, Lucy Kithinji, Joy Mwende, Laura Oyiengo, Martin Sirengo, Caroline E. Boeke

**Affiliations:** 1 Kenya Medical Research Institute, Nairobi, Kenya; 2 National AIDS and STIs Control Program, Ministry of Health, Kenya, Nairobi, Kenya; 3 Independent Researcher, New York, New York, United States of America; Hôpital Bichat-Claude Bernard, FRANCE

## Abstract

**Objectives:**

Kenya is one of the first African countries to scale up a national HIV viral load monitoring program. We sought to assess program scale up using the national database and identify areas for systems strengthening.

**Methods:**

Data from January 2012 to March 2016 were extracted from Kenya’s national viral load database. Characteristics of 1,108,356 tests were assessed over time, including reason for testing, turnaround times, test results, treatment regimens, and socio-demographic information.

**Results:**

The number of facilities offering viral load testing increased to ~2,000 with >40,000 tests being conducted per month by 2016. By March 2016, most (84.2%) tests were conducted for routine monitoring purposes and the turnaround time from facility-level sample collection to result dispatch from the lab was 21(24) [median (IQR)] days. Although the proportions of repeat viral load tests increased over time, the volumes were lower than expected. Elevated viral load was much more common in pediatric and adolescent patients (0-<3 years: 43.1%, 3-<10 years: 34.5%, 10-<20 years: 36.6%) than in adults (30-<60 years: 13.3%; p<0.001).

**Conclusions:**

Coverage of viral load testing dramatically increased in Kenya to >50% of patients on antiretroviral therapy (ART) by early 2016 and represents a relatively efficient laboratory system. However, strengthening of patient tracking mechanisms and viral load result utilization may be necessary to further improve the system. Additional focus is needed on paediatric/adolescent patients to improve viral suppression in these groups. Kenya’s national viral load database has demonstrated its usefulness in assessing laboratory programs, tracking trends in patient characteristics, monitoring scale-up of new policies and programs, and identifying problem areas for further investigation.

## Introduction

National HIV programs in resource-limited settings historically have used CD4 cell count, a marker of immune status, to monitor patient response to HIV antiretroviral therapy (ART). However, viral load (VL) measurement of plasma HIV concentration is a more direct measure of response to treatment. VL testing, which has been widely available in high-income countries for many years, is used to assess viral response to therapy and identify patients who may be failing treatment. The benefit of VL is that it is able to detect treatment failure earlier and more accurately than CD4 testing, enabling healthcare workers to give patients the care they require through more extensive adherence counselling or switching to second line drugs. Yet VL testing has traditionally been relatively expensive and required intensive infrastructure and human resources, creating barriers to scale-up.

Despite these challenges and concerns about feasibility in resource-limited settings,[1] a national VL program was first established in Kenya in 2012 with growth over subsequent years. Kenya was well-placed to be among the first countries in sub-Saharan Africa to build such a program given the country’s large HIV epidemic (1.5 million people living with HIV as of 2015),[2] high coverage of patients on ART (900,000 patients as of 2016) [3] and strong national HIV early infant diagnosis (EID) program. Initially, VL testing was prioritized for patients with suspected virologic failure, but starting in 2014 Kenyan guidelines recommended routine testing, defined as a VL test 6 months and 12 months after ART initiation and annually thereafter, for patients with undetectable viral loads. Patients with elevated VL (defined as ≥1000 copies/mL) should receive follow-up per national guideline algorithms.[4]

The program was designed to include an electronic data management system for patient monitoring, including patient demographic details and regimen information. Facility-level data feed into a national laboratory information system (LIS) database managed by the National AIDS & STI Control Programme (NASCOP) and are available publically via a national dashboard, an interactive computer interface tool that graphically presents program indicators. [5] Laboratory program staff and NASCOP staff use this dashboard to monitor lab delays, identify gaps in coverage, and highlight any relevant program issues, as well as monitor changes to the country’s programs, including changes to national guidelines. Data are available broken down by facility.

The aim of this analysis was to track the scale-up of Kenya’s VL program from 2012 to 2016 and identify areas for further investigation and systems strengthening using data captured in the national database.

## Methods

### Program description

VL tests have been available free of charge at all public health facilities in Kenya since 2012. Most (>90%) health facilities offering ART now use this service for patients at subsidized rates. Blood samples and patient demographic and treatment information, including patient identification number at the health facility, are sent to national laboratories on a data requisition form through a courier system. (Sample types included fresh plasma, used at some health facilities located within a short walk/drive of testing laboratories, frozen plasma, dried blood spots, and dried blood spot capillary—infants only.) The laboratories use various platforms (Abbott *m*2000 systems and Roche Cobas Ampliprep/Cobas TaqMan, as described previously[6, 7]) to determine VL. Test results and patient information are entered manually into electronic information systems at each laboratory, which are combined into the national database.

Changes have been made to Kenya’s HIV guidelines over the analysis timeframe.[8–10] In 2012, adults and adolescents were eligible for antiretroviral therapy if they had a CD4 count less than 350 cells/μL (or WHO stage 3 or 4 or TB co-infection); the CD4 threshold was higher for children 2–12 years, and all children under 2 years were eligible for treatment. Option B+ (treatment for all pregnant women) was introduced and piloted in some sites. Viral load testing was targeted and conducted for confirmation of treatment failure in patients with a decline in CD4 count or clinical symptoms suggestive of failure. In 2014, all children under 10 years became eligible for treatment, Option B+ was scaled up nationally, and all others with CD4<500 cells/μL were eligible for treatment. Routine VL monitoring was adopted for those on treatment; those with an unsuppressed/elevated VL (defined as ≥1000 copies/mL in national guidelines) were due to receive a repeat test 3 months later, following adherence intensification interventions. A second VL in that timeframe was considered to confirm treatment failure and necessitate a change to a second line treatment regimen.

As of 2012, the preferred first line regimens for adults and adolescents was TDF+3TC+EFV, TDF+3TC+NVP, AZT+3TC+EFV, or AZT+3TC+NVP. For infants and children the preferred first line was ABC+3TC+LPV/r if NVP-exposed and <10 kg, ABC+3TC+NVP if NVP-unexposed and <10kg, or ABC+3TC+NVP or ABC+3TC+EFV if >10kg. The recommended second line for adults and adolescents was AZT+3TC+LPV/r, AZT+3TC+ATV/r, TDF+3TC+LPV/r or TDF+3TC+ATV/r depending on the first line regimen. In 2014, the preferred first line for children <3 years was ABC+3TC+LPV/r, the preferred first line for children 3–10 and adolescents <35 kg was ABC+3TC+EFV, and the preferred first line for older adolescents and adults was TDF+3TC+EFV.

### Study population and data analysis

For this retrospective analysis, data were extracted in April 2016 on 1,139,620 VL samples collected between January 2012 and March 2016. We set implausible values to missing, including: ART initiation dates prior to January 1, 1996 (n = 3,528), as ART was not available in Kenya prior to this date; ages listed as 0 years (n = 108,007), as “0” indicated missing age information at many facilities; ages listed as 100+ years (n = 33); age and time on ART where these values were incompatible with each other (n = 1,090); age where patients listed as pregnant with age<9 years and listed as on an adult or unknown regimen (n = 48); test justification for those listed as pregnant, age<9 years, and on a pediatric regimen (n = 43). Duplicates and invalid test results were excluded from the analytic sample. Samples from the same patient were identified where the same facility, identification number at the facility, sex, and ART initiation date were listed; however, given that we could not unequivocally confirm samples from the same patients, limited longitudinal analysis was conducted.

The primary analysis was descriptive, including the number and percentage of tests with characteristics including reason for testing, treatment regimens, and socio-demographic information, median (IQR) turnaround times, and the percentage of elevated VLs (defined as ≥1000 copies/mL, per national and WHO guidelines). Turnaround times were calculated from VL sample collection to laboratory receipt, from laboratory receipt to processing, and from processing to laboratory dispatch. Information on dates of receipt of result by facilities and patients were not available in the database. Duration on ART was calculated from ART initiation date to sample collection date.

Analyses were conducted in Stata version 13. As a secondary analysis, we described characteristics of patients undergoing routine testing in 2015–2016 after 6+ months on ART. Groups with a high proportion of elevated VL were identified using univariate and multivariate logistic regression accounting for clustering by health facilities using the vce() function. The missing indicator method was used to account for missing data. Statistical significance was defined as p<0.05.

### Ethical approval

This study was approved by the Scientific Steering Committee and Ethical Review Committee of the Kenya Medical Research Institute (KEMRI SERU Protocol SSC No. 1066).

## Results

### Analytic sample

**Fig 1:**
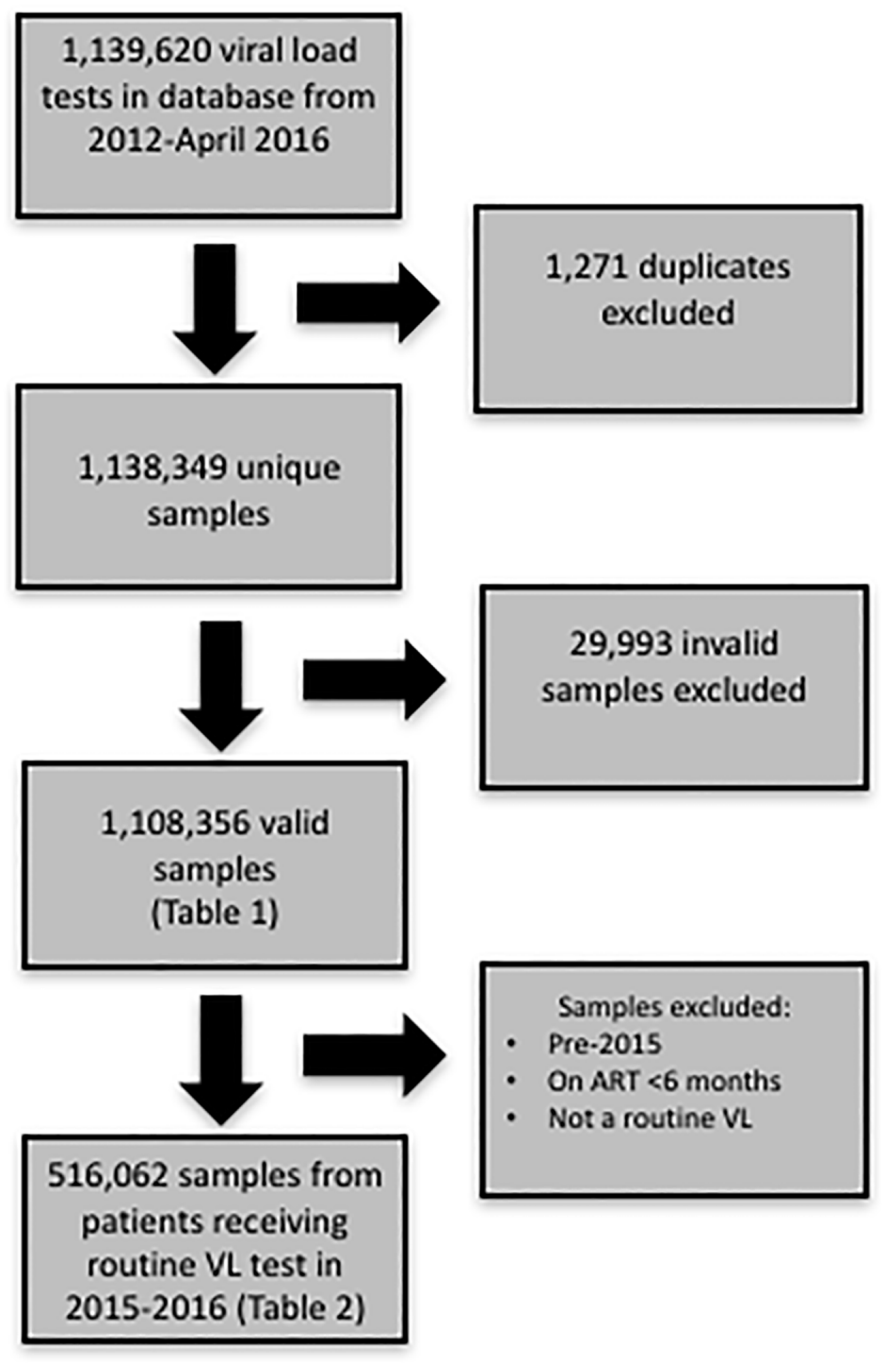
Flow Chart of samples included in the analysis.

Fig 1 describes the analytic samples included in this dataset. From the initial 1,139,620 tests in the dataset, 1,271 duplicate tests were excluded. 29,993 tests were invalid, primarily due to improper sample collection technique. After excluding these tests, there were 1,108,356 viral load samples in this dataset.

### Test characteristics

The average number of viral load tests conducted per month increased from 1,191 in 2012 to over 40,000 by 2015–2016; there was evidence that testing was beginning to plateau as of 2015–2016 (Table 1). The number of sites offering VL testing increased from 722 sites in 218 districts in 2012 to approximately 2,000 sites in over 300 districts by 2015–2016. The number of districts and sites offering testing increased when comparing first quarter data from 2015 to first quarter data (the data that were available at the time of analysis) from 2016. The number of laboratories conducting testing increased from 5 to 9 by 2016. The majority (60.3%; N = 668,692) of tests were frozen plasma, followed by DBS (30.5%; N = 338,244). Lab turnaround times increased in 2014 as the program was dramatically scaled up but decreased in 2015–2016. By 2016, the median (IQR) time from sample collection to results dispatch from the laboratory was 21 (24) days; the majority of the delays were from sample receipt at the lab to sample processing [8 (20) days], followed by sample transport to the lab [6 (8 days)].

**Table 1:** Characteristics [N/%/median (IQR)] of samples in the viral load database over program scale-up.

Variable	2012	2013	2014	2015	2016 (January-March)^a^	Total
**Test characteristics**						
Total number of tests	**14,292**	**55,075**	**294,988**	**623,101**	**120,900**	**1,108,356**
Average number of tests per month	1,191	4,590	24,582	51,925	40,300	21,732
Number of sites offering testing	722	1,041	1,943	2,583	1,997	2,876
Number of districts offering testing	218	276	304	314	304	318
Number of laboratories conducting testing	5	7	8	9	9	9
Sample type						
Fresh plasma	26.8%	16.3%	7.5%	10.1%	2.8%	9.1%
Frozen plasma	33.4%	61.5%	65.6%	57.3%	65.9%	60.3%
DBS	39.1%	21.8%	26.9%	32.6%	31.3%	30.5%
Missing	0.7%	0.4%	0.0%	0.0%	0.0%	0.0%
Days from collection to lab receipt	5 (9)	6 (9)	6 (11)	7 (12)	6 (8)	6 (11)
Days from lab receipt to lab dispatch	8 (11)	7 (13)	33 (81)	21 (27)	8 (20)	20 (35)
Days from test result to lab dispatch	3 (6)	2 (7)	3 (8)	2 (3)	2 (4)	2 (4)
Total days from collection to lab dispatch	21 (24)	22 (28)	55 (88)	36 (35)	21 (24)	36 (42)
Justification for test						
Routine test	3.5%	8.5%	57.8%	72.1%	84.2%	65.6%
Confirmation of treatment failure	12.9%	10.0%	1.1%	1.0%	1.2%	1.7%
Clinical or immunological failure	40.4%	29.3%	7.0%	1.3%	1.0%	4.7%
Other	15.8%	11.3%	10.8%	5.0%	3.7%	6.9%
Missing	27.4%	40.9%	23.3%	20.5%	9.8%	21.2%
Test status^b^						
First test in database	99.2%	97.6%	97.1%	93.8%	90.3%	94.6%
Second or greater test from a patient who tested previously	0.8%	2.4%	2.9%	6.2%	9.7%	5.4%
Viral load						
Undetectable	81.4%	80.5%	80.5%	81.4%	82.8%	81.2%
Detectable but suppressed	1.4%	1.6%	1.4%	1.5%	1.3%	1.4%
Elevated (≥1000 copies/mL)	17.2%	18.0%	18.1%	17.2%	15.9%	17.3%
**Patient characteristics**						
Sex						
Male	35.5%	35.5%	30.4%	28.5%	30.5%	29.7%
Female	60.5%	58.1%	58.7%	61.5%	65.9%	61.0%
Missing	4.0%	6.4%	10.9%	10.0%	3.6%	9.3%
Age						
0-<3 years	0.4%	0.4%	0.4%	0.4%	0.4%	0.4%
3-<10	3.0%	3.7%	4.4%	4.6%	4.2%	4.4%
10-<20 years	6.0%	6.4%	5.2%	5.9%	5.9%	5.8%
20-<30 years	11.9%	10.8%	11.3%	12.2%	11.7%	11.8%
30-<60 years	66.0%	65.0%	64.2%	60.8%	59.8%	61.9%
60+ years	4.1%	4.1%	4.6%	4.5%	4.4%	4.5%
Missing	8.6%	9.5%	9.9%	11.6%	13.5%	11.2%
Time on ART						
0-<6 months	3.6%	2.7%	2.8%	2.8%	2.4%	2.7%
6 months-<1 year	3.4%	2.4%	2.8%	7.7%	9.7%	6.3%
1-<2 years	9.1%	6.5%	4.8%	8.3%	12.8%	7.8%
2-<5 years	29.0%	25.4%	13.4%	19.3%	25.2%	18.8%
5-<10 years	13.1%	14.5%	10.9%	15.2%	20.7%	14.6%
10+ years	0.1%	0.3%	0.6%	1.2%	2.5%	1.1%
Missing	41.8%	48.3%	64.7%	45.6%	26.7%	48.7%
Current ART regimen						
NNRTI-based regimens (adults)	40.6%	40.9%	68.7%	72.9%	82.9%	70.9%
PI-based regimens (adults)	1.8%	2.6%	3.0%	2.8%	3.7%	3.0%
NNRTI-based regimens (peds)	1.3%	2.3%	3.5%	3.7%	3.4%	3.5%
PI-based regimens (peds)	0.1%	0.3%	0.4%	0.5%	0.7%	0.5%
None	5.0%	1.7%	0.7%	0.2%	0.2%	0.4%
Other	42.4%	22.0%	3.7%	4.7%	2.2%	5.5%
Missing	8.9%	30.3%	20.0%	15.3%	6.9%	16.3%
Province						
Nairobi and Central	20.9%	19.9%	20.1%	20.6%	25.9%	21.0%
East: Coast, Eastern, and North Eastern	24.1%	9.1%	7.3%	15.3%	16.6%	13.1%
West: Rift Valley, Western, Nyanza	52.1%	47.9%	63.3%	63.0%	56.6%	61.5%
Missing	2.9%	23.1%	9.3%	1.1%	0.9%	4.4%

^a^ Test/site/district numbers in 2016 were slightly lower than 2015, likely due to not all samples from those months being uploaded to the database at the time of data extraction.

^b^Determined by considering samples with the same facility, ID number at the facility, sex, and ART initiation date as coming from the same patient. Note that given that some patients may have transitioned to other facilities or the recordkeeping may have been inconsistent over time, these numbers likely have some degree of inaccuracy. DBS: Dried blood spot. ART: Antiretroviral therapy. NNRTI: non-nucleoside reverse transcriptase inhibitor. PI: protease inhibitor.

Routine viral loads became increasingly dominant over time, representing 84.2% (N = 101,745) of 120,900 tests in January-March 2016. The percentage of samples linked to a patient who tested previously was 0.8% (N = 112 of 14,292) in 2012 compared to 9.7% (N = 11,716) in January-March 2016. The percentage of elevated VL tests was 17.2% (N = 2,454) in 2012, 18.1% (N = 53,327 of 294,988) in 2014, and 15.9% (N = 19,277) in January-March 2016 (2012 vs. 2016 comparison: p = 0.03); a small percentage of tests were suppressed but detectable.

### Patient characteristics

Across time, the majority of tests were from women (61.0%; N = 676,528). The age distribution of testing over time was relatively constant, with 0.4% (N = 4,553) being children <3 years, 4.4% (N = 49,071) children 3-<10 years, 5.8% (N = 63,853) adolescents 10-<20 years, 11.8% (N = 130,802) young adults 20-<30 years, 61.9% (N = 685,968) adults 30-<60 years, and 4.5% (N = 49,753) older adults 60+ years. By January-March 2016, the median (IQR) duration on ART was 3.4 (4.2) years; 2.4% (N = 2,888) of samples came from patients on ART <6 months, 9.7% (N = 11,745) were on ART 6 months-<1 year, 12.8% (N = 15,479) were on ART 1-<2 years, 26.7% (N = 32,253) were missing information on duration on ART, and the remainder of patients on ART for 2+ years. The proportion of adults on non-nucleoside reverse transcriptase inhibitor (NNRTI)-based regimens such as tenofovir disoproxil fumarate, lamivudine and efavirenz (TDF+3TC+EFV), the current recommended first line regimen, increased over time and comprised 82.9% (N = 100,247) of all samples by January-March 2016. By January-March 2016, most (56.6%; N = 68,388) of the samples came from Western Kenya (Rift Valley, Western, and Nyanza provinces), whereas 25.9% (N = 31,362) came from Nairobi and Central Kenya and 16.6% (N = 20,049) came from Eastern Kenya (Coast, Eastern, and North Eastern provinces).

### Routine VL in 2015–2016

After excluding patients on ART for less than 6 months and adults on PI-based regimens, there were 516,062 routine tests conducted from January 2015-March 2016 (Table 2). Males (N = 154,446) were approximately half as likely to get a VL test compared to females (N = 331,166) and elevated VL was slightly more common in males, even after adjusting for other factors (17.8%; N = 27,423 vs. 15.8%; N = 52,390; p<0.001). Elevated VL was most common in children <3 years (43.1%; N = 947/2,195), followed by adolescents 10-<20 years (36.6%; N = 10,794/29,489) and children 3-<10 years (34.5%; N = 8,771/25,428); in comparison, 13.3% (N = 43,185/325,348) of adults 30-<60 years had an elevated VL and the percentage was even lower in adults 60+ years (10.5%; N = 2,596/24,700; all comparisons with adults p<0.001). The odds of elevated VL in children <3 years was nearly 5 times the odds in adults 30-<60 years in multivariable models (OR: 4.99, 95% CI: 4.51–5.52). Elevated VL was more common in samples from patients who had been on ART for 1 to 5 years compared to those on ART 6 months to 1 year (17.0%; N = 29,610/174,417 vs. 13.0%; N = 6,275/48,111; p<0.001) and in samples from Eastern (16.4%; N = 13,680/83,358) and Western (17.4%; N = 58,928/338,840) Kenya compared to Nairobi and Central Kenya (12.5%; N = 10,990/87,777; p<0.001).

**Table 2:** Characteristics [N (%)] of samples from patients receiving a routine viral load test after 6+ months on first-line^a^ treatment in 2015–2016 and associations with elevated VL (≥1000 copies/mL)^b^.

Variable	Total N	N ≥ 1000 copies/mL (%)	Univariate OR (95% CI)	p-value	Multivariate OR (95% CI)	p-value
	**516,062**	**16.4%**				
Sex						
Male	154,446	17.8%	1.15 (1.12–1.18)	<0.001	1.08 (1.05–1.10)	<0.001
Female	331,166	15.8%	Ref		Ref	
Missing	30,450	15.7%	0.99 (0.87–1.14)	0.91	0.99 (0.90–1.10)	0.90
Age						
0-<3 years	2,195	43.1%	4.96 (4.49–5.48)	<0.001	4.99 (4.51–5.52)	<0.001
3-<10 years	25,428	34.5%	3.44 (3.29–3.60)	<0.001	3.32 (3.18–3.47)	<0.001
10-<20 years	29,489	36.6%	3.77 (3.60–3.95)	<0.001	3.77 (3.61–3.95)	<0.001
20-<30 years	65,944	18.6%	1.50 (1.45–1.55)	<0.001	1.52 (1.47–1.57)	<0.001
30-<60 years	325,348	13.3%	Ref		Ref	
60+ years	24,700	10.5%	0.77 (0.73–0.81)	<0.001	0.75 (0.71–0.79)	<0.001
Missing	42,958	14.0%	1.06 (0.94–1.20)	0.32	1.21 (1.12–1.32)	<0.001
Time on ART						
6 months-<1 year	48,111	13.0%	Ref		Ref	
1-<5 years	174,417	17.0%	1.36 (1.31–1.41)	<0.001	1.47 (1.42–1.53)	<0.001
5-<10 years	93,758	14.9%	1.17 (1.11–1.22)	<0.001	1.29 (1.23–1.36)	<0.001
10+ years	8,468	12.0%	0.91 (0.83–1.01)	0.08	1.13 (1.03–1.24)	0.01
Missing	191,308	17.6%	1.43 (1.34–1.52)	<0.001	1.44 (1.36–1.53)	<0.001
Province						
Nairobi and Central	87,777	12.5%	Ref		Ref	
East: Coast, Eastern, and North Eastern	83,358	16.4%	1.37 (1.22–1.54)	<0.001	1.31 (1.17–1.46)	<0.001
West: Rift Valley, Western, Nyanza	338,840	17.4%	1.47 (1.33–1.62)	<0.001	1.38 (1.26–1.52)	<0.001
Missing	6,087	16.5%	1.38 (1.27–1.50)	<0.001	1.32 (1.22–1.43)	<0.001

^a^Because the database does not explicitly label first vs. second line treatments, adults on PI-based regimens were assumed to be on second line and were excluded from this analysis.

^b^Univariate and multivariable logistic regression accounting for clustering by health facility. The multivariable model was adjusted for all factors in this table as well as year of sample collection. The missing indicator method was used to account for missing data. Bold indicates statistical significance at p<0·05. ART: Antiretroviral therapy.

### Subsequent VL testing

Of patients with a first routine VL test in 2015 that was elevated (N = 67,166), 4.1% (N = 2,769) received a second VL test by March 2016 that was linkable to the first sample based on facility, ID number, sex, and ART initiation date; 1.6% (N = 1,040) received that second test within 4 months. There certainly may have been more second VL tests performed that were not identified as subsequent tests or linked to the first sample. The median (IQR) time to second VL in those with an elevated first VL was 144 (88) days. Of those with a second test, 71.2% (N = 1,971) demonstrated virologic failure (second VL ≥1000 copies/mL). Of patients whose first test was suppressed and received a second VL by March 2016 (N = 4,704), the median (IQR) time to second VL was 175 (146) days and 7.2% (N = 338) had an elevated second test.

### Database quality

Missing data and inconsistencies in the data were observed to a varying extent, depending on the characteristic. For most characteristics, missing data decreased by 2016. Duration on ART was the characteristic most commonly missing, with 48.7% (N = 539,613) of samples missing data (down to 26.7%; N = 32,253 in early 2016). Given this, as a sensitivity analysis to assess the impact of this variable on the multivariable regression analysis, we removed time on ART from the model and the same variables shown in Table 2 remained statistically significant; as another sensitivity analysis, we ran the multivariable model using the complete case approach and Table 2 findings were again consistent. 21.2% (N = 235,046) of tests were missing test justification information, although missing data appeared to decrease to 9.8% (N = 11,894) of samples in January-March 2016. 9.3% (N = 103,012) of samples were missing information on patient sex (3.6%; N = 4,309 in January-March 2016), 11.2% (N = 124,356) were missing information on age (13.5%; N = 16,363 in 2016), and 16.3%; N = 180,437 (6.9%; N = 8,283 in January-March 2016) were missing treatment regimen. 49,563 samples were missing information on health facility and 197 samples were missing a patient identification number. Data inconsistencies included samples being listed as a routine VL but the patient was on treatment for less than 6 months and pediatric patients on adult regimens. These data quality issues represent areas for database improvement to enhance system monitoring in the future.

## Discussion

Kenya’s national VL database has allowed for tracking of program scale-up, monitoring changes to patient and treatment characteristics over time, and identification of areas for improvement. VL access (number of facilities offering VL testing given the number of people living with HIV and ART coverage) has increased since 2012, with approximately 2,000 facilities offering testing and over 40,000 samples tested per month in 2016. Turnaround times for laboratory testing increased from 2012 to 2014 but decreased to [median (IQR)] 21 (24) days by 2016. The primary source of delays appeared to be the time between lab receipt of the VL sample and lab processing of the sample, followed by transport time to the lab. The proportion of routine tests increased over time, as did the proportion of tests that appeared to be subsequent tests from the same patients. The proportion of tests that reported elevated VL decreased by 2015–2016; in these years, among patients receiving routine VL after at least 6 months on treatment, elevated VL was 16.4%. Elevated VL was more common among children and adolescents, as well as males. Samples from Nairobi and Central Kenya were slightly more likely to be suppressed than samples from elsewhere. Missing data and inconsistencies in the database were observed, although some of these issues appeared to be decreasing over time. Follow-up tests appeared to be uncommon among patients with a first elevated test; virologic failure was very high in those who had a second test.

Kenya’s program has demonstrated an impressive scale-up of testing volumes and the database has demonstrated its utility in tracking growth and monitoring problem areas. As the program grew through 2014, VL testing turnaround times increased greatly, as sample transportation systems were streamlined and laboratories adapted to respond to increased demand. Turnaround times from sample collection to laboratory result dispatch decreased to approximately 3 weeks (not including time to results delivery), most of which was laboratory processing time. Given the volume of samples and the size of the country, these processing times may be considered reasonable, but it may be possible to decrease turnaround times further still in subsequent years, such as through review of laboratory workflow and identification of inefficiencies. Introduction of point-of-care VL testing, which allows VL tests to be processed at the facility within hours, is likely to dramatically decrease turnaround times and may reduce the burden on centralized laboratories for VL testing, further improving systems efficiencies.

In this national-level analysis, viral suppression among routine testers in 2015–2016 was similar to what has been described in other studies[11, 12] but below the aims of the UNAIDS 90-90-90 targets. Patient suppression has appeared to increase over time as better drugs have become available and as routine VL monitoring has increased. As ART programs are expanded in Kenya to treat all patients regardless of CD4 count, and as dolutegravir (Tivicay, GlaxoSmithKline, Brentford, United Kingdom), a new, more potent and more durable drug, becomes part of the preferred first line regimen, the percentage of suppressed patients may increase further. However, strong adherence counselling programs and patient support programs to reduce loss to follow-up will be important to ensure that patients who initiate treatment remain in care. Median duration on ART at time of VL test was relatively long (3.4 years), which may be explained by routine VL monitoring starting relatively recently, meaning that many patients were only receiving VL tests for the first time after years on treatment. However, in this database, there were relatively few tests that could be identified as coming from the same patient and very few patients receiving a test for confirmation of treatment failure given the number of patients with elevated VL. This could be due to issues with patient linking/tracing in the facility/laboratory data systems and/or indicate that VL testing and failure management guidelines are not yet fully being followed. Improvements in facility data systems and better understanding of adherence to patient failure management guidelines and reasons for nonadherence are needed to strengthen the VL monitoring systems and improve patient care.

In this database, elevated VL was much more common in children and adolescents than in adults, consistent with previous studies in Kenya and elsewhere in sub-Saharan Africa.[13–17] Children generally have higher VLs than adults[18] due to lower immune competence and the time it takes for VL to decrease from the high levels at diagnosis.[19] Many studies show that when HIV drugs are working, patients should be fully suppressed within six months,[20–22] but it is unclear whether this standard applies to young children. In this dataset, the median time on ART for patients under 3 years was 1.1 years and higher in older age groups. Other potential reasons for higher VLs in this population include difficulties associated with administering medications to young children, including large tablet size, crushing behaviors which reduce drug bioavailability, non-palatability of liquid formulations, lack of appropriate formulations and fixed-dose combinations, cold chain requirements for LPV/r, potential under-dosing, and potential resistance to NVP-based regimens.[23–26] As of 2016, Kenya began procuring heat-stable LPV/r pellets, which are easier for small children to swallow and will hopefully improve pediatric adherence. The high VLs observed in adolescents supports previous findings of poorer outcomes in this age group,[27] which may be explained by non-adherence because of stigma, poor transition to adult care, and lack of social support.[28, 29] Collectively, given the greater proportions with elevated VL in these groups, paediatric and adolescent populations should be prioritized for intervention. Initial analyses of these programmatic data led to targeted interventions for these populations at poorly performing facilities in Kenya with the aim of understanding barriers improving suppression in these populations.

There were modest differences in the proportion of males and females with viral suppression. Not surprisingly, vastly more women received tests than men, likely explained by greater care-seeking behaviour in women[30] as well as the success of prevention of mother-to-child transmission (PMTCT) programs in testing pregnant women and linking them to care. In contrast, men may be more likely to be tested for HIV because poor health conditions (e.g., TB) bring them into contact with the health system, and tend to present for care at more severe disease stages.[30] Of note, the difference between males and females appeared to be strongest among youth. Male youth face distinct challenges that may be amenable to intervention [31–33]. Programs should continue efforts to support male enrolment and retention in HIV care.

There were also modest regional differences in suppression, with a higher proportion of suppressed patients in Nairobi and Central Kenya compared to Eastern and Western Kenya. This may be due to geographic differences in access to services and/or patient characteristics.

As registry-based data, our findings are limited by several factors including incomplete or inaccurate entries. Ability to confirm tests as coming from the same patients and assess repeated testing was limited; therefore, analyses were primarily cross-sectional and included some repeat tests from the same individuals. Due to the limited ability to link patients in the database, the longitudinal analysis was of limited scope and must be interpreted cautiously. There was no information on adherence or indication for treatment (e.g., tuberculosis co-infection) and the database was not directly linked to clinical or patient care data, so we could not distinguish the cause of failure (nonadherence vs. resistance) and did not assess treatment regimens in detail. It is not possible to discern from these data the extent to which the changes in viral suppression over time are due changes in the populations being tested (targeted vs. routine) vs. changes in ART formulations. Due to these limitations and the observational nature of these data, causal conclusions cannot be drawn and thus the analysis was primarily descriptive. Finally, the database does not capture private sector patients, although only a very small percentage of HIV patients are managed in this sector.

While Kenya’s VL program has grown substantially, there is room for continued growth and improvement of the quality of patient care. As countries move towards treating all patients with HIV, regardless of CD4 count, further expansion of VL testing volumes and capacity will be needed. As programs scale up, it will be crucial to put strong patient tracking and failure management programs into place to ensure that VL tests are used effectively and patients are placed on the best possible treatment. Neighbouring countries such as Malawi and Uganda are also building national databases to track their programs, which are already demonstrating their utility.[34] Building strong monitoring systems like these national databases will be important to monitor scale-up and identify areas for program strengthening.
